# Supplementary material for: Transvaginal Natural Orifice Transluminal Endoscopic Surgery (vNOTES) in Urogynecological Surgery: A Systematic Review
Source: J Clin Med. 2024 Sep 25;13(19):5707. doi: 10.3390/jcm13195707 (PMC11477206; doi:10.3390/jcm13195707)
Supplement: Supplementary file 1 [file jcm-13-05707-s001.zip › jcm-3170698-supplementary.pdf]

Systematic review

A list of fields that can be edited in an update can be found here

1. * Review title.

Give the title of the review in English

Transvaginal natural orifice transluminal endoscopic surgery (V-NOTES) in urogynecological surgery: a systematic review

2. Original language title.

For reviews in languages other than English, give the title in the original language. This will be displayed with the English language title.

English

3. * Anticipated or actual start date.

Give the date the systematic review started or is expected to start.

15/03/2023

4. * Anticipated completion date.

Give the date by which the review is expected to be completed.

15/06/2023

5. * Stage of review at time of this submission.

This field uses answers to initial screening questions. It cannot be edited until after registration.

Tick the boxes to show which review tasks have been started and which have been completed.

Update this field each time any amendments are made to a published record.

The review has not yet started: Yes

Review stage	Started	Completed
Preliminary searches	No	No
Piloting of the study selection process	No	No
Formal screening of search results against eligibility criteria	No	No
Data extraction	No	No
Risk of bias (quality) assessment	No	No
Data analysis	No	No

Provide any other relevant information about the stage of the review here.

6. * Named contact.

The named contact is the guarantor for the accuracy of the information in the register record. This may be any member of the review team.

Riccardo Lombardo

Email salutation (e.g. "Dr Smith" or "Joanne") for correspondence:

Dr Lombardo

7. * Named contact email.

Give the electronic email address of the named contact.

rlombardo@me.com

8. Named contact address

Give the full institutional/organisational postal address for the named contact.

Via Jacini 34 00191 Rome Italy

9. Named contact phone number.

Give the telephone number for the named contact, including international dialling code.

+390633778711

10. * Organisational affiliation of the review.

Full title of the organisational affiliations for this review and website address if available. This field may be

completed as 'None' if the review is not affiliated to any organisation.

Ospedale Sant'Andrea

Organisation web address:

11. * Review team members and their organisational affiliations.

Give the personal details and the organisational affiliations of each member of the review team. Affiliation refers to groups or organisations to which review team members belong. **NOTE: email and country now MUST be entered for each person, unless you are amending a published record. PLEASE USE AN INSTITUTIONAL EMAIL ADDRESS IF POSSIBLE.**

Dr Riccardo Lombardo. Ospedale Sant'Andrea
Dr Alessio Guidotti.
Dr Simone Albisinni.
Dr Riccardo Campi.
Dr Gianluca Sampogna.
Dr Laura Pellizzari.
Dr Paolo Geretto.
Dr Lorenzo Vacca.

12. * Funding sources/sponsors.

Details of the individuals, organizations, groups, companies or other legal entities who have funded or sponsored the review.

None

Grant number(s)

State the funder, grant or award number and the date of award

13. * Conflicts of interest.

List actual or perceived conflicts of interest (financial or academic).

None

14. Collaborators.

Give the name and affiliation of any individuals or organisations who are working on the review but who are not listed as review team members. **NOTE: email and country must be completed for each person, unless you are amending a published record.**

15. * Review question.

State the review question(s) clearly and precisely. It may be appropriate to break very broad questions down into a series of related more specific questions. Questions may be framed or refined using PI(E)COS or similar where relevant.

Which are the outcomes of transvaginal natural orifice transluminal endoscopic surgery (V-NOTES) in

urogynecological surgery?

16. * Searches.

State the sources that will be searched (e.g. Medline). Give the search dates, and any restrictions (e.g. language or publication date). Do NOT enter the full search strategy (it may be provided as a link or attachment below.)

The MEDLINE/PubMed, Embase, Google Scholar, Web of Science and Cochrane controlled trials databases and clinicaltrial.gov will be searched for all relevant publications in english regarding dimensions and outcomes of stone surgery (from 1995 to present).

17. URL to search strategy.

Upload a file with your search strategy, or an example of a search strategy for a specific database, (including the keywords) in pdf or word format. In doing so you are consenting to the file being made publicly accessible. Or provide a URL or link to the strategy. Do NOT provide links to your search **results**.

Alternatively, upload your search strategy to CRD in pdf format. Please note that by doing so you are consenting to the file being made publicly accessible.

Do not make this file publicly available until the review is complete

18. * Condition or domain being studied.

Give a short description of the disease, condition or healthcare domain being studied in your systematic review.

Transvaginal natural orifice transluminal endoscopic surgery in urogynecology

19. * Participants/population.

Specify the participants or populations being studied in the review. The preferred format includes details of both inclusion and exclusion criteria.

Patients undegoing urogynecological surgery

20. * Intervention(s), exposure(s).

Give full and clear descriptions or definitions of the interventions or the exposures to be reviewed. The preferred format includes details of both inclusion and exclusion criteria.

Transvaginal natural orifice transluminal endoscopic surgery

21. * Comparator(s)/control.

Where relevant, give details of the alternatives against which the intervention/exposure will be compared (e.g. another intervention or a non-exposed control group). The preferred format includes details of both inclusion and exclusion criteria.

Any

22. * Types of study to be included.

Give details of the study designs (e.g. RCT) that are eligible for inclusion in the review. The preferred format includes both inclusion and exclusion criteria. If there are no restrictions on the types of study, this should be stated.

Any type of study

23. Context.

Give summary details of the setting or other relevant characteristics, which help define the inclusion or exclusion criteria.

Any study evaluating the use of V-NOTES

24. * Main outcome(s).

Give the pre-specified main (most important) outcomes of the review, including details of how the outcome is defined and measured and when these measurement are made, if these are part of the review inclusion criteria.

Success

Measures of effect

Please specify the effect measure(s) for you main outcome(s) e.g. relative risks, odds ratios, risk difference, and/or 'number needed to treat.

Hazard ratios (HR) will be used to estimate the size of intervention differences where available. For binary/dichotomous/categorical benefit or harm outcomes, we will use risk ratios (RR) or odds ratios (OR) where available. We will use mean difference (MD) or standardised mean difference (SMD) for continuous outcomes with corresponding 95% confidence intervals (CIs).

For prognostic factor studies, the discriminative effect is defined by the area under the receiver operator curve (AUC) with its corresponding 95% confidence interval (CI). The standard error of the AUC can be calculated with Newcombe Method.

25. * Additional outcome(s).

List the pre-specified additional outcomes of the review, with a similar level of detail to that required for main outcomes. Where there are no additional outcomes please state 'None' or 'Not applicable' as appropriate to the review

Surgical time

Hb drop

VAS score

Hospitalization

Complications according to Clavien Classification system

Measures of effect

Please specify the effect measure(s) for you additional outcome(s) e.g. relative risks, odds ratios, risk difference, and/or 'number needed to treat.

Hazard ratios (HR) will be used to estimate the size of intervention differences where available. For binary/dichotomous/categorical benefit or harm outcomes, we will use risk ratios (RR) or odds ratios (OR) where available. We will use mean difference (MD) or standardised mean difference (SMD) for continuous outcomes with corresponding 95% confidence intervals (CIs).

For prognostic factor studies, the discriminative effect is defined by the area under the receiver operator curve (AUC) with its corresponding 95% confidence interval (CI). The standard error of the AUC can be calculated with Newcombe Method.

26. * Data extraction (selection and coding).

Describe how studies will be selected for inclusion. State what data will be extracted or obtained. State how this will be done and recorded.

Two review authors from each collaborating group will independently extract outcome data. Study characteristics will be extracted by one review author and a second review author will check data extractions for accuracy. Any disagreements will be resolved by discussion or by consulting a third review author. A standardised data extraction form will be developed and piloted before its use. In case of any incompletely reported data, study authors will be contacted.

27. * Risk of bias (quality) assessment.

State which characteristics of the studies will be assessed and/or any formal risk of bias/quality assessment tools that will be used.

The 'risk of bias' of each included study will be assessed by two review authors working independently. Any disagreements will be resolved by discussion or by consulting a third review author.

28. * Strategy for data synthesis.

Describe the methods you plan to use to synthesise data. This **must not be generic text** but should be **specific to your review** and describe how the proposed approach will be applied to your data. If meta-analysis is planned, describe the models to be used, methods to explore statistical heterogeneity, and software package to be used.

If the extracted data allows, for quantitative measures (e.g. utilities and health status values), we will pool studies to generate an estimate and associated 95% confidence intervals using the inverse variance method (random effect model) in Review Manager software. For qualitative measures, reviewers will develop initial codes based on topics present in the narrative descriptions, organize sentences and paragraphs into these codes, and categorise codes to build descriptive themes (thematic analysis). Based on identified themes, we will construct a conceptual framework to guide the organisation and presentation of results based on the content analysis.

29. * Analysis of subgroups or subsets.

State any planned investigation of ‘subgroups’. Be clear and specific about which type of study or participant will be included in each group or covariate investigated. State the planned analytic approach.

Type of surgery

30. * Type and method of review.

Select the type of review, review method and health area from the lists below.

Type of review

Cost effectiveness

No

Diagnostic

No

Epidemiologic

No

Individual patient data (IPD) meta-analysis

No

Intervention

No

Living systematic review

No

Meta-analysis

No

Methodology

No

Narrative synthesis

No

Network meta-analysis

No

Pre-clinical

No

Prevention

No

Prognostic

No

Prospective meta-analysis (PMA)

No

Review of reviews

No

Service delivery

No

Synthesis of qualitative studies

No

Systematic review

Yes

Other

No

Health area of the review

Alcohol/substance misuse/abuse

No

Blood and immune system

No

Cancer

No

Cardiovascular

No

Care of the elderly

No

Child health

No

Complementary therapies

No

COVID-19

No

Crime and justice

No

Dental

No

Digestive system

No

Ear, nose and throat

No

Education

No

Endocrine and metabolic disorders

No

Eye disorders

No

General interest

No

Genetics

No

Health inequalities/health equity

No

Infections and infestations

No

International development

No

Mental health and behavioural conditions

No

Musculoskeletal

No

Neurological

No

Nursing

No

Obstetrics and gynaecology

Yes

Oral health

No

Palliative care

No

Perioperative care

No

Physiotherapy

No

Pregnancy and childbirth

No

Public health (including social determinants of health)

No

Rehabilitation

No

Respiratory disorders

No

Service delivery

No

Skin disorders

No

Social care

No

Surgery

No

Tropical Medicine

No

Urological

Yes

Wounds, injuries and accidents

No

Violence and abuse

No

31. Language.

Select each language individually to add it to the list below, use the bin icon to remove any added in error.

English

There is not an English language summary

32. * Country.

Select the country in which the review is being carried out. For multi-national collaborations select all the countries involved.

Italy

33. Other registration details.

Name any other organisation where the systematic review title or protocol is registered (e.g. Campbell, or The Joanna Briggs Institute) together with any unique identification number assigned by them. If extracted data will be stored and made available through a repository such as the Systematic Review Data Repository (SRDR), details and a link should be included here. If none, leave blank.

34. Reference and/or URL for published protocol.

If the protocol for this review is published provide details (authors, title and journal details, preferably in Vancouver format)

Add web link to the published protocol.

Or, upload your published protocol here in pdf format. Note that the upload will be publicly accessible.

No I do not make this file publicly available until the review is complete

Please note that the information required in the PROSPERO registration form must be completed in full even if access to a protocol is given.

35. Dissemination plans.

Do you intend to publish the review on completion?

No

Give brief details of plans for communicating review findings.?

36. Keywords.

Give words or phrases that best describe the review. Separate keywords with a semicolon or new line. Keywords help PROSPERO users find your review (keywords do not appear in the public record but are included in searches). Be as specific and precise as possible. Avoid acronyms and abbreviations unless these are in wide use.

V-Notes; Urogynecology; Vaginal; Colporraphy; Endoscopy.

37. Details of any existing review of the same topic by the same authors.

If you are registering an update of an existing review give details of the earlier versions and include a full bibliographic reference, if available.

None

38. * Current review status.

Update review status when the review is completed and when it is published.New registrations must be ongoing so this field is not editable for initial submission.

Please provide anticipated publication date

Review_Ongoing

39. Any additional information.

Provide any other information relevant to the registration of this review.

40. Details of final report/publication(s) or preprints if available.

Leave empty until publication details are available OR you have a link to a preprint (NOTE: this field is not editable for initial submission). List authors, title and journal details preferably in Vancouver format.

Give the link to the published review or preprint.
